# Cardiac CT in CRT as a Singular Imaging Modality for Diagnosis and Patient-Tailored Management

**DOI:** 10.3390/jcm12196212

**Published:** 2023-09-26

**Authors:** Willem Gerrits, Ibrahim Danad, Birgitta Velthuis, Saima Mushtaq, Maarten J. Cramer, Pim van der Harst, Frebus J. van Slochteren, Mathias Meine, Dominika Suchá, Marco Guglielmo

**Affiliations:** 1 Department of Cardiology, University Medical Center Utrecht, Heidelberglaan 100, 3584 CX Utrecht, The Netherlands; 2 Department of Radiology, University Medical Center Utrecht, Heidelberglaan 100, 3584 CX Utrecht, The Netherlands; 3 Department of Perioperative Cardiology and Cardiovascular Imaging, Centro Cardiologico Monzino IRCCS, Via Parea 4, 20138 Milan, Italy; 4 CART-Tech BV, Padualaan 8, 3584 CH Utrecht, The Netherlands; 5 Department of Cardiology, Haga Teaching Hospital, Els Borst-Eilersplein 275, 2545 AA The Hague, The Netherlands

**Keywords:** heart failure, cardiac resynchronization therapy, computed tomography

## Abstract

Between 30–40% of patients with cardiac resynchronization therapy (CRT) do not show an improvement in left ventricular (LV) function. It is generally known that patient selection, LV lead implantation location, and device timing optimization are the three main factors that determine CRT response. Research has shown that image-guided CRT placement, which takes into account both anatomical and functional cardiac properties, positively affects the CRT response rate. In current clinical practice, a multimodality imaging approach comprised of echocardiography, cardiac magnetic resonance imaging, or nuclear medicine imaging is used to capture these features. However, with cardiac computed tomography (CT), one has an all-in-one acquisition method for both patient selection and the division of a patient-tailored, image-guided CRT placement strategy. This review discusses the applicability of CT in CRT patient identification, selection, and guided placement, offering insights into potential advancements in optimizing CRT outcomes.

## 1. Introduction

No improvement of left ventricle (LV) function (non-response) after cardiac resynchronization therapy (CRT) is still a present-day concern which affects more than 30% of patients [1] and is associated with significant morbidity and mortality rates [2]. Careful patient selection, informed positioning of the ventricular pacemaker lead, and optimal timing of electrode activation are important considerations for increasing the effect of CRT [1]. “Response” to CRT has, however, been expressed by numerous parameters as no definite response has been defined. Commonly, a response to CRT has been reported as a reduction of the LV end-systolic volume of at least 15%. A clinical response (e.g., reduction of heart failure or mortality) or a functional improvement class (increase in exercise tolerance or New York Heart Association (NYHA)) are other parameters used to express a response to therapy [3]. To attain the greatest possible effect, the LV lead is usually targeted to the posterolateral part of the LV, since epidemiologic studies have shown that is when the greatest benefit can be expected [4,5]. However, image-guided LV lead placement, compared to standard implantation, is associated with increased LV reverse remodeling [3]. The echocardiographic response rate may favor image guidance, although this advantage does not always directly translate to clinical response in available trials [3]. A multimodality approach using echocardiography, cardiac magnetic resonance imaging (CMR), or nuclear medicine imaging is traditionally used to visualize cardiac viability, function, and dyssynchrony [6,7]. In addition to being labor-intensive, these imaging modalities may not suit every patient. Poor acoustic windows, claustrophobia, or the presence of cardiac implantable devices limit the use of echocardiography or CMR. Technological advances, however, have driven cardiac computed tomography (CT) forward as a promising multipurpose modality. With an accuracy of left ventricular ejection fraction (LVEF) measurement similar to CMR, CT may be used for selecting patients with an LV function of less than 35% [8,9,10] and for patient follow-up after device placement. Furthermore, studies have shown contrast-enhanced CT can detect myocardial fibrosis by late iodine enhancement (LIE) with a strong correlation to late gadolinium enhancement (LGE) by CMR, which is considered the gold standard [11,12,13,14]. Lastly, CT-derived strain could be leveraged to locate the most favorable position for LV lead placement [15]. In this review, these applications of a multipurpose CT approach for CRT patients will be further discussed along with their implications.

## 2. Relevant Advances in Cardiac Computed Tomography

CT was first discovered in the 1960s, but there has been an exponential growth in the use of CT in cardiology since the introduction of 64-slice CT scanners in 2004. In recent decades, high spatial resolution, rapid image acquisition, and standardized imaging protocols producing high-quality images have contributed to its adoption into clinical practice. Next to dual-source, wide-detector CT and dual-energy CT, the photon-counting technique has recently been introduced for cardiac imaging [16]. With these hardware systems, advances have been made in spatial resolution (0.25–0.5 mm isotropy) and temporal resolution (up to 66 ms) with rapid image acquisition (240–270 ms gantry rotation) and improved contrast resolution (e.g., by increased tube-power and low kV) as well as in radiation dose reduction [15,17,18]. Essential developments in post-processing methods, such as iterative reconstruction or deep-learning image reconstruction for noise and artifact reduction, multi-energy CT-based virtual reconstructions for monochromatic (monoenergetic) images, and iodine (perfusion) maps have further enhanced image quality [19,20,21,22]. Software improvements that allow for (semi-)automated cardiac segmentation and function analysis have contributed to the adoption of cardiac CT into clinical practice [23]. As a result, a high-image quality cardiac CT scan can provide reliable information on myocardial tissue, atrial and ventricular dimensions, cardiac function, and myocardial perfusion and allow for assessment of the coronary system. With continued game-changing technological developments, further evolution of cardiac CT is to be expected.

## 3. Multipurpose Cardiac Computed Tomography for Cardiac Resynchronization Therapy Patients

Cardiac CT is a versatile imaging tool with significant potential for the CRT patient. First, it can identify heart failure patients who meet the criteria for CRT by assessing LV function and ruling out obstructive coronary artery disease. Second, it can be used to gather additional information that could influence the placement of the LV lead through techniques like CT venography, LIE-CT, and CT strain analysis. Finally, by combining these parameters, it may facilitate the development of a personalized approach for lead delivery for each patient. An overview of these CT-derived parameters is shown in Table 1 and discussed below. In practice, acquisition of these CT images is combined in an optimized protocol. Afterwards, the clinically required information can be obtained through analysis of the appropriate CT images with additional postprocessing tools.

### 3.1. Identifying Patients with Cardiac Computed Tomography That May Benefit from Cardiac Resynchronization Therapy

Cardiac CT allows for assessment of the coronary arteries and the LV volume and ejection fraction. Based on these parameters, potential CRT candidates can be identified. The guidelines’ criteria for CRT placement are shown in Table 2, in addition to optimal medical treatment [24]. It is noteworthy that in patients with a left bundle branch block, CRT is indicated to improve symptoms and reduce morbidity and mortality, whereas for patients with a non-left bundle branch block, this is for improvement of symptoms and reduction of morbidity only [24].

#### 3.1.1. Evaluation of the Coronary Artery System

Cardiac CT is already extensively used for the noninvasive evaluation of coronary anatomy. There is substantial evidence showing coronary computed tomography angiography (CCTA) can be used to rule out obstructive coronary artery disease with high certainty [25]. As such, CCTA may also be used in heart failure patients to rule out ischemic heart disease as the underlying cause of heart failure and influence the recommendation for a defibrillator functionality [24]. Indeed, meta-analysis in patients with a reduced ejection fraction has demonstrated that CCTA retains its high diagnostic accuracy and negative predictive value [26,27].

#### 3.1.2. Assessment of Ventricular Volume and Function

CMR is considered the gold standard for the determination of LV volume and function. Meta-analysis showed no significant difference between these measurements on CT and CMR [8,9,10]. In this comparison, a number of factors need to be taken into account. Due to the relatively recent advances in the field of CT, the total number of patients in the included papers is limited. Additionally, in several of the included studies, to reduce heart rate and increase temporal resolution, beta-blockers were administered prior to CT but not CMR. Furthermore, all patients in the included analysis were, at the very least, suspected of cardiac disease [8,9,10]. This demonstrates the wide clinical range of the application of CT but may limit the interpretation of more specific patient categories. Nonetheless, a Bland–Altman analysis showed a small bias [8,9] and a good correlation [10] between LVEF on CT compared to CMR. Good correlation between both modalities was also reported for end-diastolic volume (EDV), end-systolic volume (ESV), and stroke volume (SV) as well [9,10]. The mean difference between EDV, ESV, and SV on CT and CMR was 2.62 mL, 1.61 mL, and 3.21 mL [9], with a significant correlation coefficient of 0.93, 0.95, and 0.85, respectively [10]. Hence, if the appropriate images were recorded, calculation of the LVEF on a cardiac CT scan allows for identification of a potential CRT patient.

### 3.2. Comprehensive Assessment of Patients Eligible for Cardiac Resynchronization Therapy with Cardiac Computed Tomography

**Figure 1:**
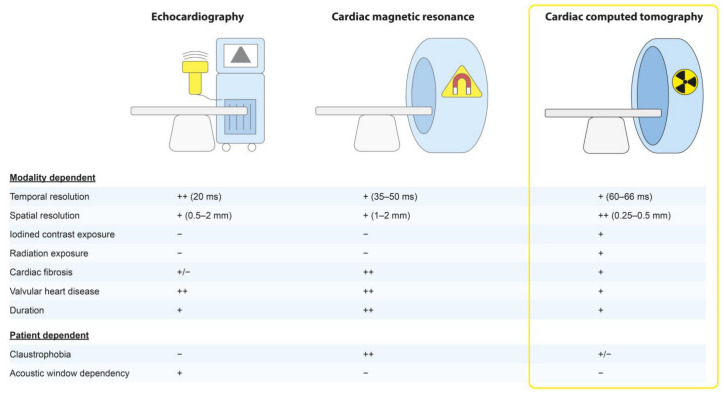
Cardiac computed tomography as the all-around imaging modality. A comparison of relevant features that differ between the imaging modalities is shown [15,17,28,29]. A “plus” indicates an association between the imaging modality and the specific factor, whereas a “minus” indicates no association. The “plus/minus” indicates a point could be made for both the presence and absence of an association.

At present, echocardiography is routinely used to evaluate CRT patients. CMR may additionally be performed, when indicated. However, CT holds the potential to become the all-around imaging modality for CRT patients. An overview of how cardiac CT compares to echocardiography and CMR is shown in Figure 1.

#### 3.2.1. Coronary Venous System

Retrograde balloon angiography is routinely acquired during CRT placement to evaluate venous anatomy and LV lead position. Variations of the coronary venous system such as anomalous insertion of the coronary sinus ostium, aberrant venous anatomy, or the presence of valves, small caliber branches, or tortuous veins can affect the implantation procedure [30,31]. This heterogeneity between the individual patients underscores the potential benefit of pre-procedural imaging. Fortunately, global venous anatomy is similar between patients with and without heart failure [32,33]. CT venography can be leveraged to assess the coronary venous system prior to LV lead placement [6,7,15,29,32,33,34,35,36,37,38,39,40]. The accuracy of CT at detecting the venous branches was shown to be, at least, equally as good as retrograde balloon angiography [35,36,37,41]. Additionally, the evaluation of valves in the coronary venous system on CT is difficult, but feasible [42].

The diameter of the venous branches is another parameter that can be measured using coronary CT [43]. Vessels where the ostium was at least 5 mm in diameter in the absence of tortuosity were considered potential target veins in one trial [41]. However, the relatively small size of the LV pacemaker lead (4 F, diameter 1.3 mm) and experience with novel delivery techniques should facilitate lead placement into vessels with a diameter smaller than 5 mm as well.

#### 3.2.2. Scar Identification

**Figure 2:**
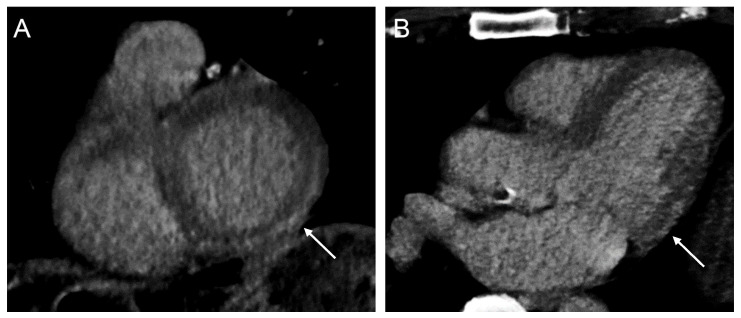
Late iodine enhancement on cardiac tomography. Images are of a 67-year-old male patient suffering from frequent monomorphic ventricular extra systoles. The arrows indicate areas of non-ischemic, subepicardial late iodine enhancement, shown in the short axis view (panel (**A**)), and the 3-chamber view (panel (**B**)).

An intermediate-to-large scar burden is detrimental for patients receiving CRT. When the LV lead is placed within the scar, outcomes have been shown to be worse [44]. Myocardial scar can be identified due to a longer retention of contrast agents in fibrotic tissue than in healthy tissue. On CMR, which is considered the gold standard technique, late gadolinium enhancement (LGE) is used for myocardial scar identification. Ironically, the first reported image that utilized late enhancement imaging to identify myocardial scarring was acquired on CT in 1976, at a time before CMR existed [45]. The iodine-based CT contrast agent is similarly retained longer in fibrotic tissue and can, as a result, be used to detect scar tissue on CT as late iodine enhancement (LIE). An example of scar tissue on LIE-CT images is shown in Figure 2. In patients with ischemic and non-ischemic heart failure, a strong correlation was found between the extent of myocardial scarring and the ability to differentiate between an ischemic and non-ischemic attenuation on LIE-CT [11,12,13,14]. However, inter-operator variability in correctly classifying LIE-CT is high and has a long learning curve [11,13,14].

Several trials have investigated the correlation between LIE-CT-based scar, LV lead location, and CRT outcome. Similar to LGE-CMR-based studies, increased echocardiographic reverse remodeling and better outcomes were reported when the LV lead was not placed within or adjacent to the scar tissue identified on the LIE-CT [29,39,44,46,47,48]. When the lead was placed within the scar tissue, this resulted in a higher mortality rate [44].

#### 3.2.3. Extracellular Volume

Another parameter that can be used to express myocardial injury is by quantification of extracellular volume (ECV). Pathological conditions that are accompanied by an expansion of the extracellular space (comprising of both the interstitial and intravascular space) will cause a rise in ECV. Therefore, most infiltrative diseases (depending on the disease stage), oedema, and fibrosis will result in elevated ECV, which is associated with heart failure, diastolic dysfunction, and increased morbidity and mortality rates [41]. ECV measurement on CMR is the gold standard, but it may also be done with CT. ECV on CT relies on the same properties as ECV on CMR by measuring the attenuation of the myocardium before and 5–7 min after administration of the contrast agent [49]. Similar to CMR, CT ECV can then be calculated with the formula:

$$ECVct=\left(1-Hematocrit\right)*\frac{∆Humyocardium}{∆Hublood}$$

ECV assessed on CT has a good inter-observer reproducibility [50,51,52,53,54,55] and a good correlation to ECV measured on CMR in patients with valve disease and different cardiomyopathies [51,52,53,54,55,56,57,58,59]. CT-derived ECV was significantly higher in patients with heart failure with a preserved ejection fraction, ischemic, and non-ischemic cardiomyopathy than in controls [60,61,62,63]. Healthy myocardium could also be distinguished from ischemic and non-ischemic myocardium based on CT-derived ECV [53,62,64]. CT-based ECV could even be determined in patients with a cardiac implantable electronic device [65]. However, many of these observations have been in non-CRT patients. Therefore, the benefit of ECV calculation in CRT patients is still unknown. Although an inverse correlation between CRT outcome and concordance of the LV lead with ECV seems likely, this still needs to be properly investigated. Furthermore, whether ECV has any additional value over LIE in relation to ventricular lead location and CRT outcome remains to be seen.

#### 3.2.4. Strain Measurement

Strain is a valuable parameter that provides information on cardiac dyssynchrony, which is especially relevant for CRT patients. The most commonly used methods to determine strain are speckle tracking echocardiography (STE) and CMR feature tracking. However, strain can also be determined on cine CT images. When CT strain is compared to other modalities, CT strain shows a fair-to-good correlation to STE and CMR [66,67,68]. Furthermore, CT-derived strain is not a static value but can improve after adequate treatment [69] and has prognostic value [70,71,72].

Strain can be expressed as a global value to inform on overall cardiac function, or as regional strain which provides information on local myocardial function and serves as a parameter of dyssynchrony. Based on the temporal difference to maximum regional strain, the latest activated segment of the LV can be identified and targeted for lead placement [15]. Previous studies have shown that CMR strain can be used to identify this latest activated region mechanically [73]. Targeted lead placement within this area is associated with improved reverse remodeling compared to data from literature [6,73]. Furthermore, CT-based regional strain differences can be used to detect scar in the CRT patient where infarcted regions showed reduced (relative) strain values [74].

**Figure 3:**
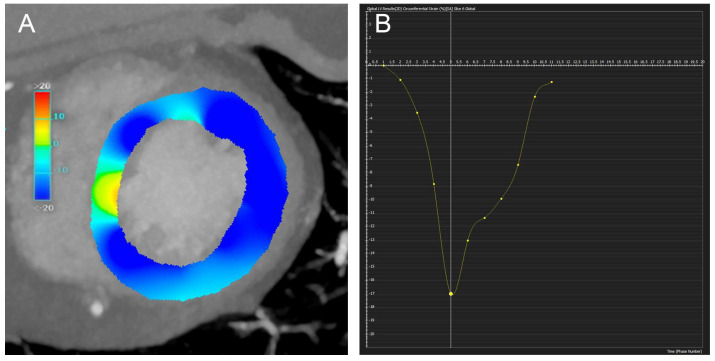
Computed tomography based strain. The circumferential strain measurement of the left ventricle (panel (**A**)), with the corresponding strain curve (Y−axis) over time (frame number, X−axis) (panel (**B**)).

Strain analysis in CRT patients has been largely limited to CMR. However, the CMR results and CT-based strain in other cardiac patients is interesting and warrants investigation of CT-based strain in CRT patients. An example of CT-based global circumferential strain measurement with corresponding strain curves is shown in Figure 3.

Even though data is scarce, CT strain is also feasible in patients with a cardiac pacemaker and can be used to assess mechanical dyssynchrony in these patients [28,75]. Patients that require an upgrade from a right ventricular pacemaker to a biventricular CRT device may also benefit from targeted LV lead placement based on CT strain. Such guided placement would be more difficult with CMR in these specific patients due to imaging artifacts and safety concerns.

#### 3.2.5. Phrenic Nerve Identification

Phrenic nerve stimulation during LV pacing is one of the reasons for selecting a different LV lead location than intended. On CT images, the pericardiophrenic bundles, containing the phrenic nerve along with the pericardiophrenic artery and vein, can be located and taken into account when selecting the optimal LV lead position [6,76,77]. This may help guide LV lead placement and reduce procedure time.

### 3.3. Cardiac Computed Tomography Guided Cardiac Resynchronization Therapy

#### 3.3.1. Guided Left Ventricular Lead Placement

The benefit of knowledge of the coronary venous system prior to implantation remains controversial and is limited by the amount of recent data. There have been reports that show a significant reduction in fluoroscopy duration at the cost of a higher total radiation dose (including the CT scan), with fewer catheters used and less contrast exposure [38,41]. However, another randomized controlled trial contradicted these findings and saw no significant reduction in procedure time or fluoroscopy duration [29].

When knowledge of the coronary venous system is supplemented with functional parameters, the value of the coronary venous anatomy becomes more apparent. Retrospective analysis of a small number of CRT patients showed that some of the non-responders had other suitable venous branches over a potentially beneficial area [39]. Prior knowledge and LV lead placement in this area could have possibly made these patients CRT responders.

The benefit of CT in CRT patients was shown in 18 patients where CT was used to guide an upgrade to CRT. By using a novel algorithm that holds a close relation to circumferential strain, the myocardial stretch in each segment was determined. When the final LV lead position was within the predetermined target, the patients showed a greater composite clinical response rate [28]. Furthermore, during CRT implantation, several other cardiac veins were cannulated with a pacing wire as well. When stimulating inside the optimal target, patients had a greater acute hemodynamic response measured by invasive pressure volume loops than when stimulated in scar tissue [28].

**Figure 4:**
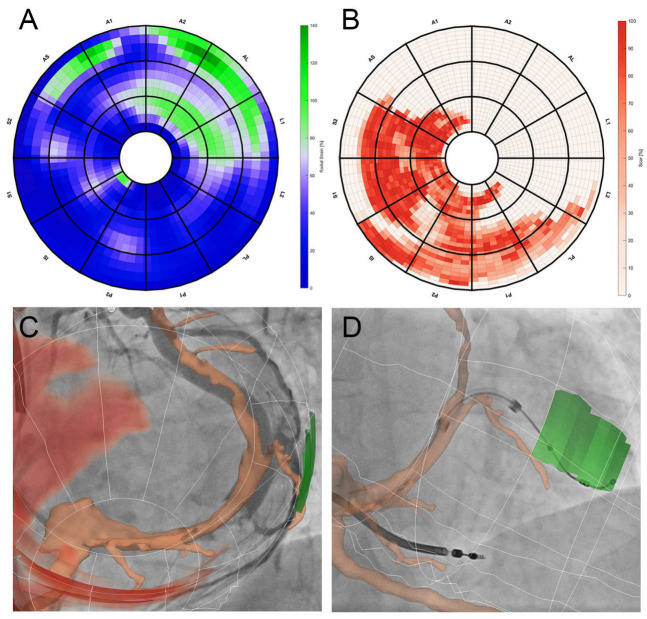
CT-based live-guided CRT placement. The maximum radial strain at the end of the cardiac cycle is located in the anterolateral segment (AL) of the left ventricle as is shown on the bulls-eye plot (panel (**A**)), and the scar is located in the septal-posterolateral (S2-PL) part of the myocardium (panel (**B**)). These areas are combined with the CT venogram to create a 3D rendering of the bulls-eye plot (with the optimal target in green and scar in red) and the coronary veins, which are projected on top of the angiography image during CRT implantation (panel (**C**)). The final lead position is within the target area, outside of the scar (which is omitted for clarity in this recording) (panel (**D**)).

Strategies have also been developed that provide “live” guidance during implantation. An example of such a model is shown in Figure 4. Live guidance was achieved based on the fusion of processed images with fluoroscopy in patients receiving CRT.

Only a few reports have been published on live-guided CRT implantation based on pre-procedural CT images. In one study, just the CT-derived coronary venous system was available. During the procedure, the implanting physician had an overview of all coronary branches which was fused with the live angiography [78]. Additionally, in the second reported study, the optimal target was selected based on the latest activated region (identified by wall motion or strain) in the American Heart Association (AHA) model [41]. On top of that, in the last study, the location of the scar was also taken into account when selecting the optimal target [15]. The coronary veins traversing and adjacent to the selected target were also defined based on the CT. Both the optimal AHA target along with the coronary veins were overlain on the angiography during implantation to facilitate the live guidance [15,41]. Even though all of these trials have shown proof that CT-guided lead placement is possible, due to the small number of patients included, the results are still ambiguous. The CT-based guidance led to a significant reduction in the total fluoroscopy time but an increase in the total radiation dose in one study [41]. Others have shown a greater acute hemodynamic response based on invasive pressure-volume loops when pacing within the predefined target than when outside of this area. Unexpectedly, a reduction of LV end-systolic volume of more than 15% only occurred in 60% of patients. This may have been the result of the ability to place the lead within the target in only 83% of patients due to coronary sinus angulation, phrenic nerve stimulation, and the size of the coronary vein being too small [15]. Patients that were undergoing an upgrade from a right ventricular pacemaker to CRT were also included. Nonetheless, after proof of concept in these small studies, larger trials are needed.

#### 3.3.2. Cardiac Computed Tomography to Determine the Method of Left Ventricular Lead Delivery

Proceeding with endovascular implantation seems logical when a target and corresponding epicardial vein have been identified. However, when there is no coronary vein near the optimal target or when there is no target at all, other strategies like conduction system pacing (CSP) or a surgical lead placement should be considered [79]. This is in line with the guideline that recommends a similar approach, as class IIa indication, when coronary sinus lead implantation has failed [24].

#### 3.3.3. Follow-up after Guided Cardiac Resynchronization Therapy

After CRT implantation, the response to the therapy needs to be monitored. In patients with poor echocardiographic windows and a CRT implant that can cause image artifacts on CMR, a CT scan seems the best option. The feasibility of CT-based follow-up in patients after CRT placement has already been shown [79].

## 4. Implications

### Financial and Safety Considerations

The mean cost of a single CRT device, including implantation, ranges from approximately €11,000 to €16,600 depending on the presence of a defibrillator function [80]. Image-guided LV lead placement positively affects response rate, but comes at a higher cost than the current standard of care. However, in a cost-effectiveness estimation, it was found that image-guided lead placement could save between €300–€20,000 per patient [81]. Additionally, a CT scan is cheaper than a CMR scan [65], further driving the cost effectiveness towards CT-guided lead placement. On top of that, a CT scan is typically faster with shorter waiting lists than a CMR scan [29,82]. This reduces the time between patient selection and CRT implantation, potentially limiting the deterioration of heart failure while awaiting implantation.

Despite the advantages of the multipurpose CT approach, radiation exposure remains a constant concern. However, a significant reduction in radiation exposure from CCTA has been seen over time. Between 2007 and 2017, the mean radiation dose dropped by 78% [83]. With new scanners and scanning protocols, strain analysis is feasible with a total effective dose of 1.78–2.8 mSv with a temporal resolution of 66 ms or 17 frames/cardiac cycle [23,84]. Multi-segment reconstruction of cardiac cine CT can also be used to reduce the radiation dose [85]. When only end-systolic strain is required, the effective dose can be as low as 1.7 mSv, one study showed [23]. Prospective ECG-triggered CTA, high-pitch scanning, iterative reconstruction, and the use of artificial intelligence are other strategies for reducing radiation exposure [21].

The other safety concern is contrast exposure, which can be as much as 120 mL for an entire cardiac CT protocol [15,28]. According to local guidelines, patients with impaired kidney function require extra preparation and monitoring for contrast scans that contain iodine. However, there is evidence that a lower contrast dosage does not affect ECV calculations [86]. With the continuing development of CT technology, further reductions in radiation and contrast exposure are likely to improve patient safety. Whether these techniques can be applied in evaluating the CRT patient needs to be investigated.

## 5. Limitations and Future Perspective

The reviewed literature shows promising developments that enable the application of CT in new fields like CRT. However, other imaging modalities such as CMR and echocardiography already have a more established role in these patients. Consequently, there is more data available on these imaging modalities than CT. As a result, standard values have been defined for these established imaging modalities. Furthermore, technical differences between CT and these gold-standard imaging modalities make comparison inherently more challenging. Fortunately, CT is catching up, with strain and ECV already being investigated with CT. However, this fast development is also a limitation of the wider application of CT, especially the use of CT in non-specialized centers and the requirement of up-to-date CT systems or post-processing tools. As a result, large prospective trials are lacking. In time, with more widespread application of these techniques, like strain and ECV, comparison should be possible in larger patient groups.

Additionally, with the adoption of CSP in patients with dyssynchronous heart failure, the developments within the field of cardiac resynchronization also need to be considered. Even though initial results may point toward a benefit of CSP over conventional CRT placement [87,88], larger comparative trials (that include image-guided CRT placement) are still needed. Furthermore, pre-procedural planning or guidance during CSP device implantation are other avenues that need to be explored as well.

## 6. Conclusions

Based on the reviewed literature, CT is a multipurpose tool that has the potential to be used for the identification of the appropriate patient for CRT, as well as treatment planning based on the evaluation of multiple patient-specific aspects. Integration of this information into the implantation procedure may enable a live, guided, patient-tailored CRT placement strategy to maximize therapy effect and prognosis.

## Figures and Tables

**Table 1:** Overview of CT studies useful for CRT.

CT Study	Purpose
Cardiac	Left ventricular dimension and function: baseline (and follow-up)
Coronary	Coronary artery and venous system anatomy and patency
Late phase	Myocardial fibrosis delineation (LIE) and extracellular volume (ECV)
Strain *	Mechanical dyssynchrony

CRT: cardiac resynchronization therapy; CT: computed tomography; LIE: late iodine enhancement; ECV: extracellular volume. * Not a separate scan but attained by post-processing.

**Table 2:** Summary of the 2021 European Society of Cardiology (ESC) guideline recommendations for cardiac resynchronization therapy (CRT).

Patient Sub-Type with an Indication for CRT	Class Recommendation
Left ventricular ejection fraction < 35% with a left bundle branch block with a QRS duration of:	
• More than 150 ms	I
• 130–149 ms	IIa
Left ventricular ejection fraction < 35% with a non-left bundle branch block with a duration of:	
• More than 150 ms	IIa
• 130–149 ms	IIb

Source: 2021 ESC Guidelines on cardiac pacing and CRT [24].

## Data Availability

No new data were created or analyzed in this study. Data sharing is not applicable to this article.
